# Intrapancreatic accessory spleens in African swine fever infection of wild boar (*Sus scrofa*)

**DOI:** 10.3389/fvets.2023.1306320

**Published:** 2023-12-12

**Authors:** Néstor Porras, Blanca Chinchilla, Antonio Rodríguez-Bertos, José Á. Barasona, Aleksandra Kosowska, Esther Vázquez-Fernández, Pedro J. Sánchez-Cordón, José M. Sánchez-Vizcaíno

**Affiliations:** ^1^VISAVET Health Surveillance Centre, Complutense University of Madrid, Madrid, Spain; ^2^Department of Animal Production, Faculty of Veterinary Medicine, Complutense University of Madrid, Madrid, Spain; ^3^Department of Internal Medicine and Animal Surgery, Faculty of Veterinary Medicine, Complutense University of Madrid, Madrid, Spain; ^4^Department of Animal Health, Faculty of Veterinary Medicine, Complutense University of Madrid, Madrid, Spain; ^5^Pathology Department, Animal Health Research Center (CISA), National Institute of Research, Agricultural and Food Technology (INIA-CSIC), Madrid, Spain

**Keywords:** intrapancreatic accessory spleen (IPAS), wild boar, low virulence isolate, African swine fever virus (ASFV), highly virulent isolate, histopathology, immunohistochemistry

## Abstract

Intrapancreatic accessory spleen (IPAS) is one of the most frequent congenital splenic anomalies in humans; however, studies in veterinary medicine are scarce. This study aimed to describe the macroscopic, histopathological and immunohistochemical features of 11 suspected cases of IPAS in wild boar piglets of 3–4 months old. Seven of the 11 animals were immunised with a low virulence isolate of African swine fever virus (ASFV) and subsequently challenged with a highly virulent ASFV isolate (LVI-HVI group). The remaining four animals were exclusively infected with a highly virulent isolate of ASFV (HVI group). Grossly, lesions comprised focal or multifocal reddish areas of variable shape, located on the surface of the pancreatic tail or within the parenchyma. Histological and immunohistochemical studies (anti-CD79 and CD3) confirmed the presence of IPAS in eight of the 11 cases. IPAS shared the same histological structure and alterations as those observed in the original spleen. The immunohistochemical study against ASFV revealed the presence of VP72+ cells in both the spleen and IPAS of seven of the eight piglets. The results of this study describe for the first time the presence of IPAS in ASFV infection of wild boar (*Sus scrofa*) regardless the isolate and suggest that the infection may induce the development of ectopic splenic tissue due to an increased demand for phagocytic cells from the reticuloendothelial system. However, further studies are needed to understand the immunological mechanisms that trigger the formation of these accessory organs.

## 1 Introduction

The spleen is the largest lymphoid organ in the body located in the left cranial abdomen and responsible for blood filtering and immunity among other functions. Congenital anomalies of the spleen are rare in animals and include duplications, absence of the spleen (asplenia/aplasia) or its development (hypoplasia), displacements (ectopia), fissures and the presence of one or more accessory spleens (1).

Accessory spleens are foci of healthy splenic tissue found separately from the main body of the spleen due to the failure of the splenic anlage fusion (2). Accessory spleens are usually few and are commonly located at or near the splenic hilum, the gastrosplenic ligament and the tail of the pancreas (3–5). The description of intrapancreatic accessory spleen (IPAS) is a frequent phenomenon in humans that poses no clinical significance (4, 6). However, the occurrence of IPASs in animals is poorly documented in the literature, with only a few cases described in dogs and cats (5, 7, 8), domestic pigs (9, 10), rabbits (3, 11–13), marine mammals (14–17) and non-human primates (18).

Grossly, IPASs appear as firm, well-demarcated, brown-to-dark red round nodules, and can be found both in the right and left lobes of the pancreas (1, 8). Microscopically, IPASs are structurally identical to the normal spleen, consisting of red and white pulp, but the presence of poorly formed or deficient white pulps and the absence of trabecular structures has been previously described in human accessory spleens (19). Therefore, the presence of accessory spleens poses a diagnostic challenge, requiring differentiation from other alterations such as haemorrhage, haematoma, haemal node, splenopancreatic fusion and endocrine or vascular tumors (16, 20, 21).

Previous studies have shown that accessory spleens are able to grow under certain stimulus (22). Thus, an increased frequency of accessory spleens has been associated with certain inherited haematologic diseases in humans (autoimmune haemolytic anemia, idiopathic thrombocytopenic purpura, “secondary hypersplenism,” Gaucher’s disease, hereditary spherocytosis, etc) (22); but an association between induced haemolytic anemia and accessory spleens has also been observed in rabbits (13). Several diseases, including lymphoma, leukemia, ITP and haemosiderosis can also involve IPAS to the same extent as the spleen (23). Furthermore, it has been shown that both spleen and ectopic splenic tissue undergo the same alterations (6).

African swine fever (ASF) is a lethal haemorrhagic disease, notifiable to the World Organisation for Animal Health (WOAH) (24). It affects domestic pigs and wild boar (*Sus scrofa*), and the causative agent is a large, complex, double-stranded DNA virus, and it is the single member of the *Asfarviridae* family of the genus *Asfivirus* (25). African swine fever virus (ASFV) induces the formation of haemorrhagic and necrotic lesions on the surface of the pancreas (26). These lesions have a similar appearance to IPAS, so they could be misdiagnosed during experimental or field studies. Therefore, histological and immunohistochemical evaluation is essential to differentiate the origin of the lesions (16).

To the best of our knowledge, this is the first report describing the presence of IPAS in wild boar (*Sus scrofa*) and associated with ASF infection. The aim of this study is to characterize suspected cases of IPAS in wild boar by macroscopic, histological and immunohistochemical study (CD79/CD3), carrying out a differential diagnosis study. Furthermore, we associate their formation and reactivity with the presence of ASFV by immunohistochemical detection of the viral protein VP72.

## 2 Materials and methods

### 2.1 Animals and samples

Samples of suspected intrapancreatic accessory spleens (IPAS) were obtained from wild boars aged 3–6 months old and weighing 10–25 kg, which was tested previously negative for the following main porcine pathogens in the region: Aujeszky virus, *Mycobacterium bovis, Mycoplasma hyopneumoniae* and porcine circovirus type 2. The thirteen wild boar piglets used in this study belonged to two previous experiments conducted under biosafety level 3 (BSL-3) conditions at the VISAVET Health Surveillance Centre of the Complutense University of Madrid (26, 27). Those experiments were carried out following European, national, and regional regulations and approved by the Ethic Committee of Comunidad de Madrid (reference PROEX 124/18).

**Figure 1:**
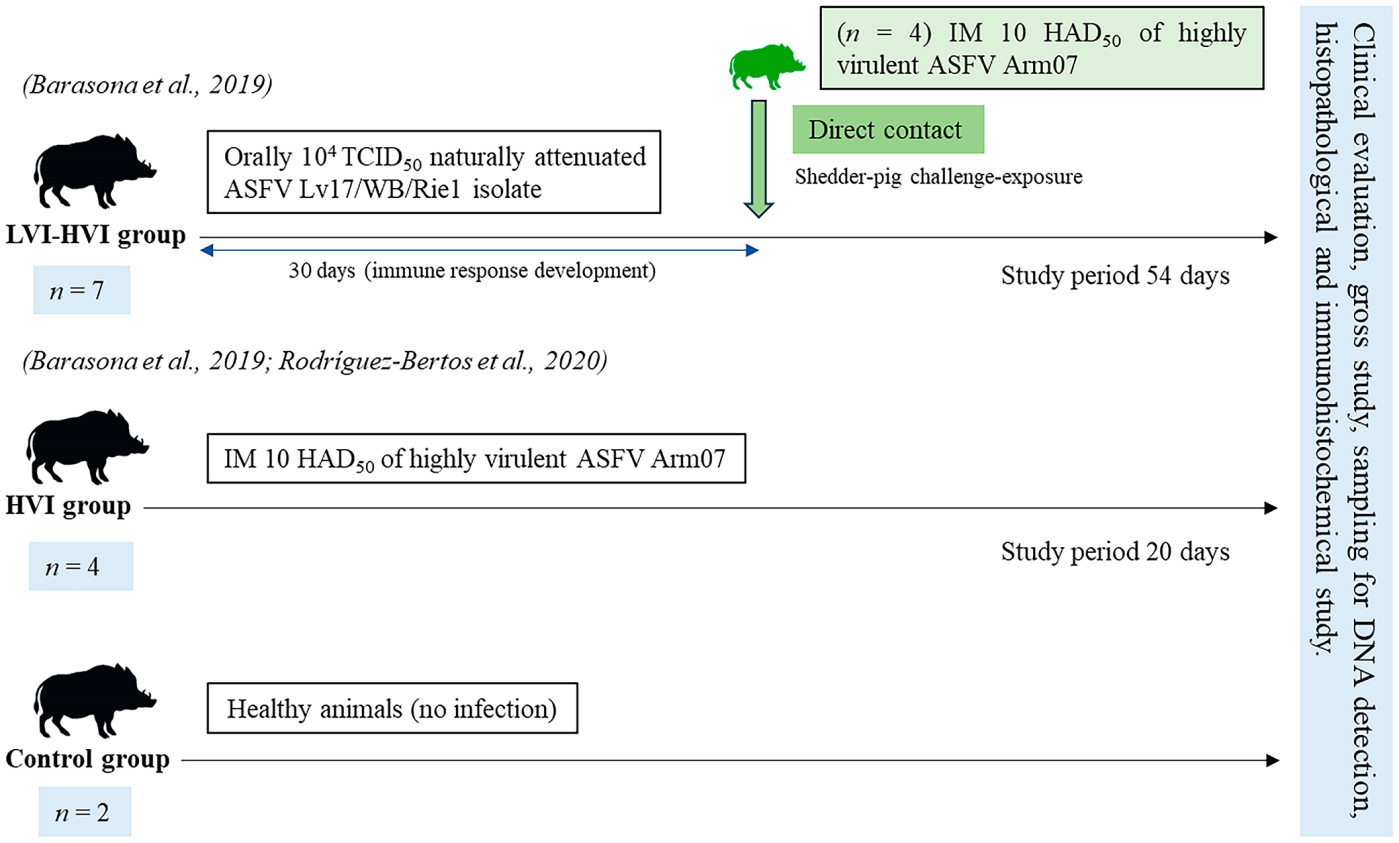
Scheme of the study design including the three groups (LVI-HVI, HVI and control group), from prime inoculation to the end of the experiment (7, 11, 15, 20, 27 and 54 days post-infection).

Seven of the thirteen animals were immunised with 10^4^ TCID_50_ (amount of virus causing cytopathic effects in 50% of infected cultures) of a naturally attenuated ASFV isolate (Lv17/WB/Rie1) and intramuscularly challenged with 10 HAD_50_ (amount of virus causing haemadsorption in 50% of infected cultures) of a highly virulent ASFV isolate (Arm07) (LVI-HVI group) (27). Four of the thirteen animals were intramuscularly inoculated with 10 HAD_50_ of ASFV Arm07 isolate (HVI group), and the remaining two animals were not infected by any of the ASFV isolates (control group) (26). The experimental design is shown in Figure 1.

### 2.2 Clinical evaluation

Daily monitoring of animal’s health status was performed by wildlife-specialist veterinarians and using a video-camera active 24 h a day. The evolution of ASFV infection was evaluated following a quantitative clinical score (CS) described by Cadenas-Fernández et al., (28). This CS is based on the evaluation of nine clinical parameters (rectal temperature, behavior, body condition, skin alterations, ocular/nasal discharge, joint welling, respiratory symptoms, digestive symptoms, and neurological symptoms) and their measurement from 0 to 4 according to the degree of severity. Fever was defined as a rectal temperature above 40.0°C. All clinical observations were daily recorded, except body temperature to minimize animal handling and stress.

Humane endpoints were established for all animals and were based on the presence of a CS greater than 18 and the appearance of severe clinical signs of disease such as fever, behavior, body condition, respiratory and digestive symptoms for more than two consecutive days (28). In addition, animals showing an unacceptable health status according to veterinary criteria were euthanized before reaching the above predefined humane endpoint.

### 2.3 Sampling and ASFV DNA detection

Whole blood samples in ethylenediaminetetraacetic acid (EDTA) were collected from each animal once a week throughout the complete study. Viral DNA was extracted from 200 μL of each sample using the High Pure Template Preparation Mix Kit (Roche Diagnostics GmbH, Mannheim, Germany) according to the manufacturer’s instructions. The detection of the ASFV DNA in blood samples was performed using the Universal Probe Library (UPL) real-time PCR (qPCR) recommended by the World Organisation for Animal Health (OIE) and the protocol previously described (29). Positive qPCR results were determined by identifying the threshold cycle value (Ct) at which reporter dye emission appeared above the background within 40 cycles. Positive and negative controls were used in DNA extraction and in qPCR.

### 2.4 Necropsy and histopathological study

Animals were necropsied and post-mortem evaluation was performed. Macroscopic changes observed in experimentally ASF-infected wild boars were evaluated according to previous protocols (26, 30). A wide variety of tissue samples from various organs were taken (26), fixed in 10% neutral formalin and routinely processed for histopathological examination. After fixation, samples were trimmed, dehydrated (Citadel 2000 Tissue Processor, Thermo Fisher Scientific, Waltham, MA), and embedded in paraffin (Histo Star Embedding Workstation, Thermo Fisher Scientific) following standard procedures. Each tissue sample block was sectioned (Finesse ME + Microtome, Thermo Fisher Scientific) and stained with hematoxylin–eosin (HE) (Gemini AS Automated Slide Stainer, Thermo Fisher Scientific). Selected sections of the IPAS were additionally stained with Masson’s trichrome (MT) for the detection of collagen fibres. Finally, samples were mounted in glass slides (CTM6 Coverslipper, Thermo Fisher Scientific) and evaluated for histopathological alterations under a Leica DM2000 microscope (Leica Microsystems, Wetzlar, 162 Germany).

### 2.5 Immunohistochemical study

Paraffin sections placed on positively charged glass slides were deparaffinized in xylene, rehydrated and subsequently antigenic retrieval was performed. These steps were carried out by the Epredia PT module Deparaffin and Heat Induced Epitope Retrieval (HIER). Endogenous peroxidase was blocked by immersing the samples in a 3% hydrogen peroxide in methanol solution (Panreac Química S.L.U.) for 15 min. Then, the samples were incubated with 2.5% Normal Horse Serum (ImmPRESS ® VR Horse AntiMouse/AntiRabbit IGG Polymer Kit, Vector Laboratories) for blocking (RTU) for 1 h. Afterwards, the slides were incubated overnight at 4°C with the primary antibodies detailed in Table 1 (DAKO, Glostrup, Denmark; Thermo Fisher Scientific, Waltham, MA, USA; Abcam, Cambridge, UK; Ingenasa, Madrid, Spain). Positive and negative controls were included in each batch of slides. For negative controls, the primary antibody was omitted and substituted by tris-buffered saline. A spleen from a wild boar infected with the Arm07 ASFV isolate was used as a positive control. After the night, the secondary antibody was added (ImmPRESS ® VR Horse AntiMouse/AntiRabbit IGG Polymer Kit, Peroxidase; Vector Laboratories) and incubated for 1 h. For the revealing process, peroxidase was used (ImmPACT ® NovaRED ® Substrate Kit Peroxidase). Afterward, the samples were counter-stained with hematoxylin (Gemini AS Automated Slide Stainer, Thermo Fisher Scientific).

**Table 1:** List of antibodies used in the immunohistochemistry study.

Antibody	Type	Host	Dilution	Company
Anti-CD3	Polyclonal	Rabbit	1:100	DAKO
Anti-CD79	Monoclonal	Mouse	1:50	DAKO
Anti-VP72 (18BG3) ASF	Monoclonal	Mouse	1:100	Ingenasa

The histopathological and immunohistochemical evaluation for IPAS detection and characterization has been based on a lymphocytic composition and trabecular structure. Thus CD3 (T cells) and CD79 (B cells) were used to characterize the phenotype lymphocytes. To detect the presence of ASFV, a semiquantitative assessment of cells immunolabeled for ASF viral antigen (protein VP72-18BG3) was performed and were evaluated as follows: immunolabeled mononuclear cells were counted in 5 adjacent, non-overlapping fields under a high-power field (HPF) magnification (400×). A score from 0 to 4 was assigned to each sample: (0) no presence of immunolabeled cells; (1) 1–10 immunolabeled cells; (2) >10–25 immunolabeled cells; (3) >25–100 immunolabeled cells; (4) >100 immunolabeled cells.

## 3 Results

### 3.1 Clinical evaluation

Animals within LVI-HVI group (*n* = 7) showed no relevant clinical signs, except fever during the viremia (27). In addition, only one vaccinated animal in the HVI-LVI group (Case No. 7) did not survive the challenge, presenting a clinical picture like that observed in animals of the HVI group. Clinical signs of animals in the HVI group (*n* = 4) were characterized by increased body temperature, decreased alertness, walking difficulties, generalized erythema, slight ocular discharge, and digestive symptoms such as presence of mucus in stools and sporadic vomiting (26). Only one animal in the HVI group (Case No. 8) had no clinical signs compatible with ASF disease. On the other hand, control animals (*n* = 2) showed no clinical signs.

### 3.2 Viral loads (blood)

Animals in the LVI-HVI group showed sporadic peaks of viremia during immunization and co-infection with the virulent ASFV isolate. All animals within that group were negative (Ct = 40) at 54 days post-challenge (dpc) against ASFV. On the other hand, animals in the HVI group presented high viral loads in blood (Ct = 25.18 ± 7.37), while no viremia was detected in animals belonging to the control group (Ct = 40).

### 3.3 Gross and histopathological examination

**Figure 2:**
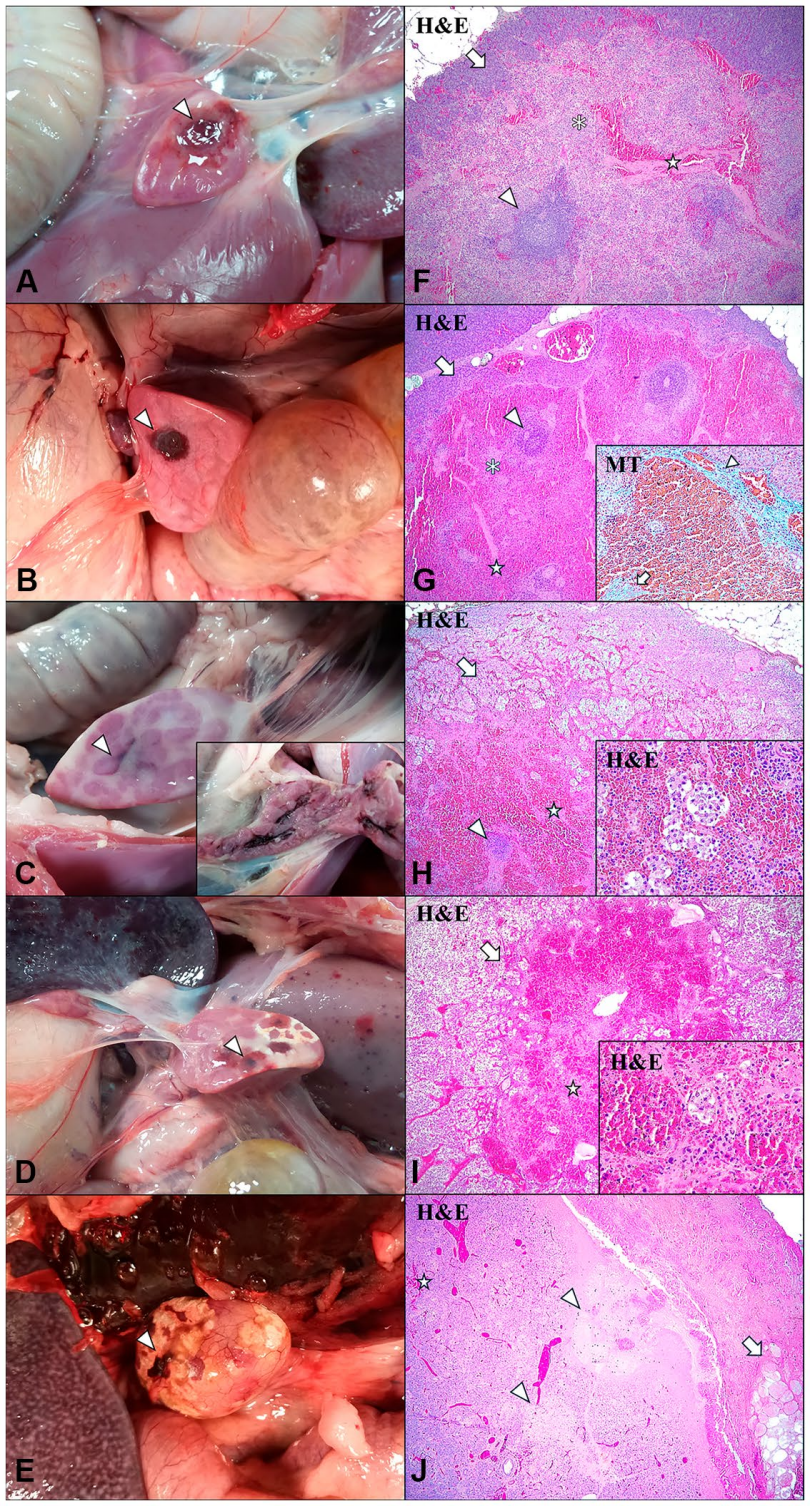
Intrapancreatic accesory spleen (IPAS) and differential diagnosis in wild boar. Macroscopic evaluation **(A–E)** and histopathologic evaluation **(F–J)**. **(A)** Case No. 3. Well-demarcated, focal, light red and round nodule localized in the pancreatic tail surface (arrowhead). **(B)** Case No. 8. Well-demarcated, focal, dark red and round nodule localised in the pancreatic tail surface (arrowhead). **(C)** Case No. 6. Non well-demarcated, multifocal, interlobular irregular dark red area in the pancreatic tail surface (arrowhead). Inset: interior of the pancreatic parenchyma, observing interlobular dark red areas. **(D)** Case No. 7. Non well-demarcated, multifocal, dark red areas (arrowhead) with white foci compatible with necrosis in the pancreatic tail surface. **(E)** Case No. 10. Non well-demarcated, focal, irregular dark red area (arrowhead) with white-yellowish areas compatible with necrosis in the pancreatic tail surface. **(F)** Case No. 3. Histological features of the IPAS, including lymphoid follicular structures (arrowhead), red pulp-like areas and splenic strings (asterisk), and trabeculaes (star). IPAS was well demarcated and separated from the pancreatic parenchyma (arrow). H&E, 4x. **(G)** Case No. 8. Histological study of the IPAS revealed lymphoid follicular structures (arrowhead), red pulp-like areas, showing marked red pulp hyperemia, and splenic strings (asterisk), and trabeculaes (star). IPAS was well demarcated and separated from the pancreatic parenchyma (arrow). H&E, 4x. Inset: blue staining determined the presence of connective tissue in the partial fibrous capsule (arrowhead) and trabeculae (arrow). Masson’s thricrome (MT) stain, 20x. **(H)** Case No. 6. Histological study of the IPAS revealed marked red pulp hyperemia, small lymphoid follicles (arrowhead), trabeculae (star) and disruption of the pancreatic acini cells (arrow). H&E, 4x. Inset: disrupted pancreatic acini cells embedded in red pulp. H&E, 40x. **(I)** Case No. 7. Histological study of the IPAS revealed total depletion of lymphoid structures, observing only small trabeculae (star) and disruption of the pancreatic acini cells (arrow). H&E, 4x. Inset: disrupted pancreatic acini cells embedded in red pulp. H&E, 40x. **(J)** Case No. 11. Histological study revealed areas of necrosis of the pancreatic acini cells (arrowheads) and small foci of haemorrhages (star), surrounded by an interlobular necrosis of the adipose tissue (arrow). H&E, 4x. (Figure 2D) was previously published by our research team (26).

Macroscopic evaluation of the 11 animals suspected of having IPAS revealed the presence of single or multifocal light to dark reddish areas located on the surface of the pancreatic tail or within the pancreatic parenchyma. However, differences were observed in the shape, appearance, and location of the accessory pancreatic tissue in the animals. Thus, five animals (Cases No. 1, 2, 3, 5, 8) had a single, well-defined, round red nodule, approximately 2 cm in diameter, slightly depressed and located on the surface of the pancreatic tail (Figures 2A,B). The remaining eight animals showed multifocal irregular reddish areas on the surface of the pancreas, which on section constituted extensive intrapancreatic interlobular foci (Figure 2C). In some cases (Cases No. 7, 9, 10, 11), these multifocal irregular lesions were accompanied by yellowish white superficial areas compatible with foci of necrosis (Figures 2D,E).

The histological study confirmed that eight of the 11 suspected animals (Cases No. 1–8) displayed IPAS, one of them (Case No. 7) also displaying severe lymphoid depletion and necrotizing pancreatitis. The remaining three animals (Cases No. 9–11) showed only necrotic and haemorrhagic lesions not compatible with accessory spleens (Table 2). The IPAS of animals was characterized by the presence of histological features similar to those observed in the spleen, including white pulp-like areas consisting of lymphoid follicles and periarteriolar lymphoid sheaths (PALS), a reticuloendothelial system and red pulp-like areas. In addition, the presence of thick trabeculae subdividing the accessory splenic tissue was also noted.

**Table 2:** Details of each animal, with histopathological diagnosis and VP72-ASF immunoexpression score.

Case *N*°	Group	DPI	Histopathological diagnosis	VP72-ASF Immunoexpression score
1	LVI-HVI	54	IPAS	0
2	LVI-HVI	54	IPAS	1
3	LVI-HVI	54	IPAS	1
4	LVI-HVI	54	IPAS	1
5	LVI-HVI	54	IPAS	1
6	LVI-HVI	54	IPAS	1
7	LVI-HVI	27	IPAS & Necrotizing pancreatitis	2
8	HVI	20	IPAS	1
9	HVI	7	Necrotizing pancreatitis	2
10	HVI	15	Necrotizing pancreatitis	3
11	HVI	11	Necrotizing pancreatitis	2
12	Control	–	–	0
13	Control	–	–	0

Histologically, certain differences in the structure and cellular composition of the IPAS were observed between the different animals in each group. In Cases No. 1–4 from LVI-HVI group, the white pulp was located around an artery or arteriole, forming lymphoid follicles and PALS (Figure 2F). The secondary lymphoid follicles had a high cell density with small germinal centres and a clearly demarcated mantle zone, whereas PALS were composed of small lymphocytes. Besides, red pulp areas contained splenic cords with macrophages arranged in the form of a reticular meshwork, capillaries, a high number of erythrocytes mainly surrounding the trabeculae and numerous circulating lymphocytes within the venous sinuses (Figure 2F). Although the splenic tissue was well demarcated, there was no fibrous capsule covering the accessory spleen and therefore no separation from the pancreatic tissue was identified. Case No. 8 in HVI group presented an accessory splenic tissue of similar structure to Cases No. 1–4 in LVI-HVI group, except for the presence of hyperaemia of the red pulp and the presence of a partial fibrous capsule in the intersection between the accessory spleen and the pancreatic tissues, confirmed by Masson’s trichrome (MT) technique (Figure 2G). On the other hand, Cases No. 5–6 in LVI-HVI group showed areas of red pulp infiltrating the pancreatic tissue, with presence of embedded pancreatic acini cells. In addition, pancreatic acini cells surrounding IPAS exhibited marked degeneration, with presence of karyorrhectic and pycnotic nuclei (Figure 2H).

The histological structure of the IPAS was very different in Case No. 7 within LVI-HVI group, with total absence of white pulp and marked hyperaemia of the red pulp. The red pulp cell population included abundant circulating lymphocytes within the venous sinuses and a high number of pale cells with nuclear debris compatible with apoptotic histiocytes or tingible body macrophages (TBM). Moreover, the IPAS was partially separated from the pancreatic tissue by a thin fibrous connective tissue capsule and trabeculae emerged from the capsule and entered into the splenic parenchyma (Figure 2I). Cases No. 9–11 in HVI group showed a histological structure not compatible with IPAS, with necrosis of the pancreatic acini and surrounding adipose tissue, as well as the presence of haemorrhagic foci (Figure 2J).

A comparative histological study was performed between cases which displayed the IPAS and its original spleen. In Cases No. 1–4 within LVI-HVI group, the red and white pulp showed similar cell density, slightly larger follicular sizes and the formation of an evident germinal centre and mantle zone. The IPAS of Cases No. 5, 6 and 8 within LVI-HVI and HVI groups was very similar in structure to the cases cited above, except for the presence of moderate hyperaemia of the red pulp. In Case No. 7 of the LVI-HVI group, IPAS showed marked depletion of the white pulp with reduced size of lymphoid follicles and PALS, and severe necrosis of lymphoid cells (lymphocytolysis). Additionally, intense hyperemia and hemorrhages were observed in the red pulp, as well as coagulative necrosis of the perifollicular areas and the reticuloendothelial system accompanied by the infiltration of a large number of TBMs (see Supplementary Figure S1 for comparative histopathology between different cases of IPAS and their original spleen).

### 3.4 Immunohistochemical study

**Figure 3:**
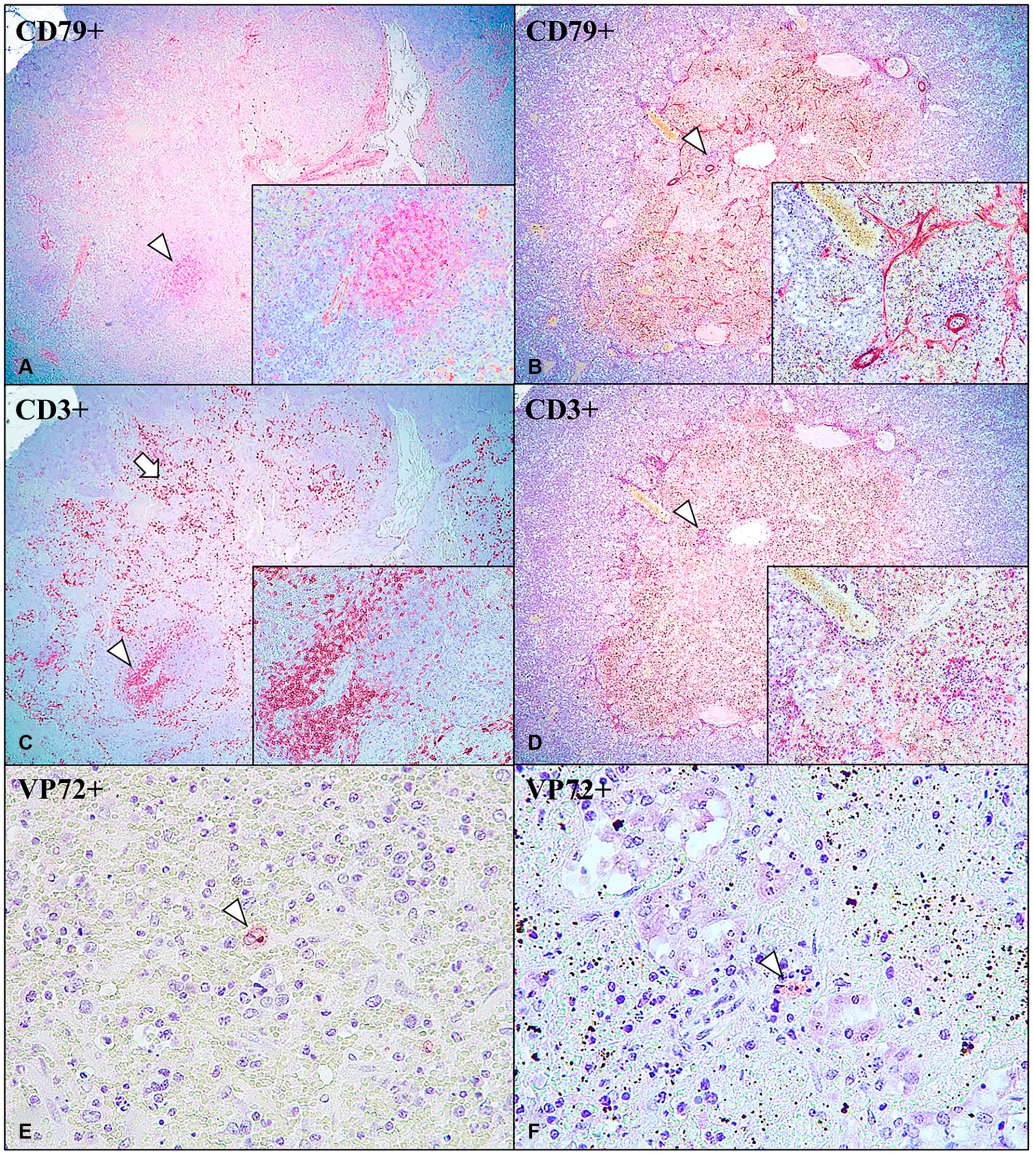
Intrapancreatic accessory spleen (IPAS) in wild boar. Case No. 3 **(A,C,E)** and Case No. 7 **(B,D,F)**. **(A)** CD79+ B cells immunoexpression in the centre of lymphoid follicle (arrowhead); anti-CD79, 4x. Inset: lymphoid follicle; 40x. **(B)** Minimal CD79+ B cells immunoexpression in lymphoid follicle (arrowhead); anti-CD79, 4x. Inset: depleted lymphoid follicle; 40x. **(C)** CD3+ T cells immunoexpression in PALS and surrounding lymphoid follicles (arrowhead), and in the circulating T cells in the venous sinuses (arrow); anti-CD3, 4x. Inset: PALS; 40x. **(D)** CD3+ T cells immunoexpression in depleted lymphoid follicle (arrowhead), and in the circulating T cells in the venous sinuses; CD3, 4x. Inset: depleted lymphoid follicle; 40x. **(E)** VP72+ macrophage inside IPAS red pulp area (arrowhead); anti-VP72, 40x. **(F)** VP72+ macrophage inside IPAS red pulp area (arrowhead); anti-VP72, 40x.

Immunohistochemical expression of CD3 and CD79 revealed the presence of a cell distribution pattern in the IPAS similar to that observed in a normal spleen. Cases No. 1–6 and 8 within LVI-HVI and HVI groups showed dense follicular aggregations of CD79+ B cells (Figure 3A) surrounded by a thin rim of CD3+ T cells (Figure 3C). PALS were composed of CD3+ cells and positive CD3 immunoexpression was also observed multifocally on circulating T lymphocytes within venous sinuses (Figure 3C). On the other hand, CD79+ B lymphocytes expression were minimally observed in Case No. 7 (Figure 3B), the only animal in the LVI-HVI group that did not survive the challenge. However, positive CD3 immunoexpression was detected on circulating T lymphocytes and lymphocytes from depleted lymphoid follicles (Figure 3D). Animals diagnosed with necrotizing pancreatitis (Cases No. 9–11 of the HVI group) and control animals (Cases No. 12–13) did not show immunostaining neither CD3 or CD79 in any of pancreatic tissue section studied.

The immunohistochemical study of the ASFV revealed that seven of the eight positive cases of IPAS (7/8; 87.5%) showed immunoreactivity to the anti-VP72 monoclonal antibody (Table 2). VP72+ cells within IPAS included mainly macrophages from the splenic cords, and secondarily, some pancreatic acini cells, characterized by diffuse brown and granular cytoplasmic staining. All animals in the LVI-HVI group displayed mild to moderate positive VP72 immunoexpression (Figure 3E), except for Case No. 1 which was negative. Positive immunolabeling of VP72 was also observed in Case No. 7 (Figure 3F). On the other hand, animals with necrotizing pancreatitis (Cases No. 9–11) showed moderate VP72 immunoexpression in mononuclear cells and pancreatic acini cells, while control animals (Cases No. 12–13) were negative (Table 2).

## 4 Discussion

The intrapancreatic accessory spleen (IPAS) has being identified as a common congenital splenic anomaly in humans, however, studies in veterinary medicine are scarce (7–9). Most of these studies do not establish the cause behind the presence of IPAS but describe its accidental finding and misdiagnosis due to its high similarity with other types of lesions (20).

Gross evaluation of wild boar tissue samples compatible with IPAS confirmed the presence of single or multiple nodular lesions mainly located in the tail of the pancreas or within the parenchyma (31). However, these well-demarcated, nodular lesions were not exclusive to IPAS in ASFV-infected wild boars, as not well-demarcated, irregular and interlobar lesions were also found in some of the animals in this study. To our knowledge, this is the first time that this irregular appearance has been described. In this sense, macroscopic findings shown here highlight the difficulty to differentiate the IPAS from other types of lesions such as hemorrhages and necrosis in pancreas.

The histological study of wild boar tissue samples compatible with IPAS confirmed the presence of both white and red pulp in eight of the 11 cases studied. Nevertheless, some typical histological structures of the spleen, such as lymphoid follicles, were absent in some animals of the study. A poorly formed or deficient white pulp in the presence of a normal appearing red pulp has been previously described in humans (19). Furthermore, we report the presence of exocrine pancreatic acini embedded in the red pulp areas of the accessory splenic tissue, a novel histomorphological feature in IPAS, only reported, in the veterinary literature, in a harbor porpoise case (*Phocoena phocoena*) (16).

Immunohistochemical result confirmed the histological features, detecting in the accessory spleens the presence of both CD3+ and CD79+ cells. CD3+ cells (T cells) were mainly located in large numbers in the periarteriolar lymphoid sheaths (PALS) and red pulp, whereas CD79+ cells (B cells) occupied the centre of the lymphoid follicles (16).

On the other hand, the presence of a fibrous capsule partially separating the adjacent pancreatic parenchyma from the accessory spleen was observed a few of the animals studied (2/8 cases). A fibrotic capsule surrounding accessory spleens has also been observed in previous studies in minipigs (9) and other animals (8, 16), suggesting that capsule formation is not related to the animal species. Regarding the presence of trabeculae, it should be noted that all wild boars in the study with confirmed presence of IPAS had trabecular connective tissue. On the contrary, most reports describe the accessory spleen as histologic structures lacking trabeculae (19). For this reason, the observation of trabeculae by histopathological evaluation (H&E), may contribute to the diagnosis of IPAS. In addition, immunohistochemical studies can also be useful for diagnosis, especially the detection of circulating CD3+ T cells, located in high numbers in the red pulp, even in those cases in which there was lymphoid depletion of the follicles. Thus, following this work routine, we confirmed 8 cases of IPAS out of the 11 suspected cases.

In this study, the same histological structures and tissue damage were observed in the IPAS and the original spleen of each ASFV-infected wild boar, suggesting that both organs underwent the same sensitivity to virus-induced damage. Thus, one of the animals in LVI-HVI group showed marked lymphoid depletion of the white pulp with the presence of TBMs in both the spleen and IPAS. Contrary, in some cases, both organs revealed white pulp immunoreactivity, characterised by the formation of germinal centres composed of CD79+ B lymphocytes. These results agree with previous studies as both the spleen and accessory spleen are influenced by the same circulatory and hormonal factors, and have identical functions (6). For this reason, lesions that affect the spleen will also affect the accessory spleen (6). On the other hand, although the functionality of the IPAS could be similar to that of the spleen, previous studies of splenosis in humans have described a limited and poor protective capacity of the ectopic splenic tissue (2).

Immunohistochemical study of ASF revealed the presence of a reduced number of VP72+ cells in seven of the eight cases of IPAS (87.5%). In these cases, macrophage tropism of ASFV was observed in both the red pulp of the spleen and IPAS. These results are consistent with the spleen being one of the main target organs of ASFV (32). Therefore, the formation of germinal centres in the white pulp suggests a reaction of IPAS against ASFV infection. Nevertheless, the histopathological findings observed in the spleen and IPAS of the previously immunised LVI-HVI group animals did not correspond to the changes previously described in infections with highly virulent isolates, except for one animal in the group (Case No. 7), the only one in the LVI-HVI group that did not survive challenge. This animal presented VP72+ mononuclear cells and showed typical lesions of highly virulent ASFV infection such as marked lymphoid depletion, lymphocytolysis, splenic hyperaemia and ischemic necrosis in both the spleen and IPAS (32). As indicated above, severe alterations of the accessory spleens due to infection with highly virulent isolates of ASFV require the use of complementary immunohistochemical techniques for their correct diagnosis.

IPAS is considered a splenic malformation of congenital origin (33). Concordantly, this anomaly was observed in eight of the 11 animals in the study that belonged to two different farms with similar health status, sanitary plans, and genetic lineage; furthermore, nothing similar was found in the control animals. However, previous studies in minipigs suggest that accessory spleens can be found in animals independently of their genetic background, with individual phenotypic differences observed between genetically identical cloned pigs (9).

Furthermore, previous studies in humans have proposed that tiny undetectable remnants of splenic tissue could grow under stimulation by certain haematologic diseases to clinically detectable sizes (22). A study in rabbits treated with phenylhydrazine, an inducer of acute haemolytic anemia, confirmed the development of accessory spleens in response to haematologic stress, suggesting that accessory spleens are not exclusively a congenital anomaly (13). Besides, animals injected with syngeneic spleen cells and simultaneously treated with particulate antigens, such as homotransplantable (ascites) tumor cells or BCG mycobacteria, presented increased enhanced development of the accessory spleen (34). One of the possible explanations for this phenomenon could be an increased demand for phagocytic cells from the spleen reticuloendothelial system (13).

Macrophages play a key role in ASFV infection, with the spleen being one of the main target organs of the virus (32, 35). In line with the plausible explanation described above, the results shown here suggest that infection with attenuated strains of ASFV, used as potential vaccine candidates, could be related not only to a higher survival rate of the animals but also to the development of accessory spleens due to an increased demand for phagocytic cells provided by the splenic reticuloendothelial system and promoted by a long-term stimulation of the immune system.

In conclusion, although the diagnosis of IPAS remains difficult, histologic and immunohistochemical evaluations are essential to differentiate ectopic splenic tissue from similar histopathologic lesions. The presence of accessory spleens in chronic ASFV infection of wild boar suggests that the virus could stimulate the development and alteration of this congenital ectopic splenic tissue with immune function. Further studies are required to explain the causes triggering the appearance and/or development of accessory spleens in animals, as well as to understand their exact functionality and origin.

## Data availability statement

The original contributions presented in the study are included in the article/supplementary material, further inquiries can be directed to the corresponding author.

## Ethics statement

The animal study was approved by Ethic Committee of Comunidad de Madrid (reference PROEX 124/18). The study was conducted in accordance with the local legislation and institutional requirements.

## Author contributions

NP: Conceptualization, Data curation, Investigation, Methodology, Software, Writing – original draft. BC: Data curation, Formal analysis, Methodology, Visualization, Writing – review & editing. AR-B: Conceptualization, Resources, Supervision, Visualization, Writing – review & editing, Investigation. JB: Data curation, Methodology, Resources, Supervision, Visualization, Writing – review & editing. AK: Data curation, Methodology, Visualization, Writing – review & editing. EV-F: Methodology, Visualization, Writing – review & editing. PS-C: Resources, Supervision, Visualization, Writing – review & editing. JS-V: Funding acquisition, Project administration, Resources, Supervision, Visualization, Writing – review & editing.
