# Supplementary material for: Intrapancreatic accessory spleens in African swine fever infection of wild boar (Sus scrofa)
Source: Front Vet Sci. 2023 Dec 12;10:1306320. doi: 10.3389/fvets.2023.1306320 (PMC10754525; doi:10.3389/fvets.2023.1306320)
Supplement: Supplementary file 1 [file Data_Sheet_1.docx]

Supplementary Material
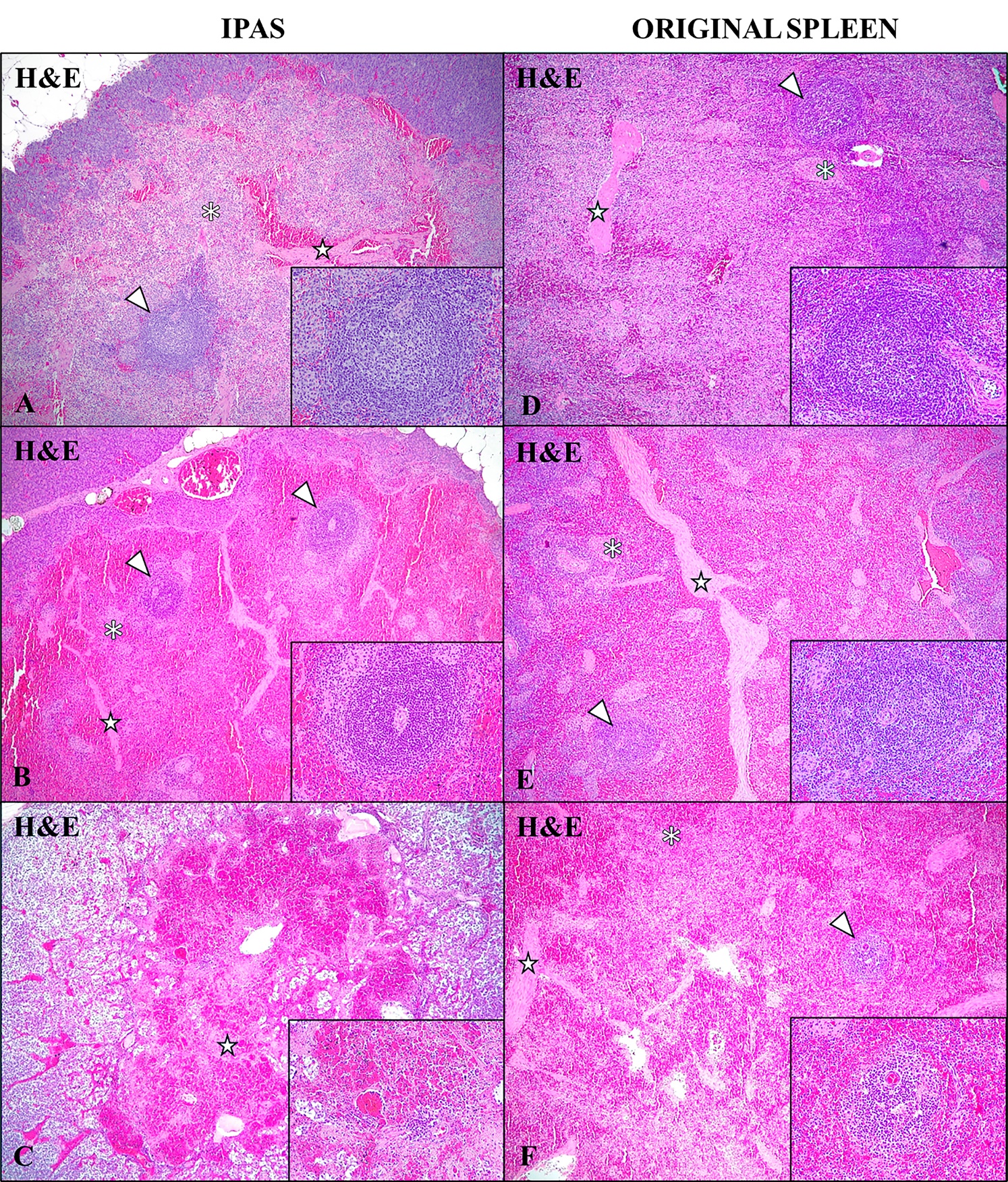


**Supplemental Figure 1.** Comparative histolopathological evaluation between IPAS (A,B,C) and original spleen (D,E,F). (A) Case No. 3. Histological features of the IPAS, including lymphoid follicular structures (arrowhead), red pulp-like areas and splenic strings (asterisk), and trabeculaes (star). H&E, 4x. Inset: lymphoid follicle, 40x. (B) Case No. 8. Histological study of the IPAS revealed lymphoid follicular structures (arrowhead), red pulp-like areas, showing marked hyperemia, and splenic strings (asterisk), and trabeculaes (star). H&E, 4x. Inset: lymphoid follicle, 40x. (C) Case No. 7. Histological study of the IPAS revealed total depletion of lymphoid structures, with only small trabeculae (star). H&E, 4x. Inset: central artery and depleted lymphoid follicle, with a minimal number of lymphoid cells, 40x. (D) Case No. 3. Histological features of the spleen, including lymphoid follicular structures (arrowhead), red pulp areas and splenic strings (asterisk), and trabeculaes (star). H&E, 4x. Inset: lymphoid follicle, 40x. (E) Case No. 8. Histological study of the spleen revealed lymphoid follicular structures (arrowhead), red pulp area, showing moderate hyperemia, and splenic strings (asterisk), and trabeculaes (star). H&E, 4x. Inset: lymphoid follicle, 40x. (F) Case No. 7. Histological study of the spleen revealed depleted lymphoid follicular structures (arrowhead), red pulp area, showing intense hyperemia, and splenic strings (asterisk), and trabeculaes (star). H&E, 4x. Inset: depleted lymphoid follicle with coagulative necrosis of the mantle zone and foci of lymphocytic necrosis in the germinal centre, 40x.
